# CircRNAs: Emerging Bladder Cancer Biomarkers and Targets

**DOI:** 10.3389/fonc.2020.606485

**Published:** 2021-01-08

**Authors:** Zhaofeng Liang, Wenhao Guo, Shikun Fang, Yue Zhang, Ling Lu, Wenrong Xu, Hui Qian

**Affiliations:** ^1^ Jiangsu Key Laboratory of Medical Science and Laboratory Medicine, School of Medicine, Jiangsu University, Zhenjiang, Jiangsu, China; ^2^ Women and Children Health Hospital of Zhenjiang, Zhenjiang, Jiangsu, China

**Keywords:** CircRNAs, bladder cancer, functions, biomarker, targets

## Abstract

Circular RNAs (circRNAs) are newly discovered intriguing RNAs due to the covalently closed loop structure, high stability, tissue specificity, and functional diversity. In recent years, a large number of circRNAs have been identified through high-throughput sequencing technology and bioinformatics methods, the abnormal expression of circRNAs are closely related to many diseases including bladder cancer (BC). CircRNAs have been proven to have several functions, such as acting as a regulator of parental gene transcription, miRNA sponge and interacting with proteins to regulate its expression. In addition, some circRNAs have been identified to encode proteins. CircRNAs have the characteristics of high abundance, high stability, wide distribution in body fluids, tissue specificity, and developmental stage specificity, which determine that circRNAs has great potential to be utilized as biomarkers for BC. Herein, we briefly summarize the biogenesis, functions and roles, and the current research progress of circRNAs in BC with a focus on the potential application for BC diagnosis, treatment, and prognosis.

## Introduction

Bladder cancer (BC) is one of the most common malignant cancers with an estimated 549,000 new cases and 200,000 deaths per year (1). Tumorigenesis processes of BC are a multicellular, multifactorial, and multistage process. Although the pathogenesis of BC are not well known, BC has been linked to tobacco smoke, parasitic infection, exposure to radiation, or chemicals and other risk factors (2, 3). Malignant transformation of normal cells, the communication of BC cells, BC stem cells, and microenvironment cells determines the initiation and progression of BC. However, the molecular mechanisms and the early diagnosis of BC have not been well elucidated. Therefore, it is urgent to explore new molecular mechanism and effective diagnosis biomarkers for BC.

Circular RNA (circRNA), a novel type of RNAs with covalently closed loop structure and lack of 3′ polyadenylated tails, are becoming a new hotspot in the field of non-coding RNAs in the recent years. CircRNAs were first discovered in the pathogenic plant viruses and considered to be the rare errors in RNA splicing. More recently, with high-throughput RNA sequencing technology, RNase R-treated transcriptomes and novel circRNA-specific bioinformatics, a large amount of circRNAs have identified in mammals, plants, fungi, worms, fish, insects, and other eukaryotes (4, 5). Since then, circRNAs have attracted widespread attention and relevant studies have been conducted on their biological properties, functions, molecular mechanisms, and potential application in clinical diagnosis and treatment.

In recent years, the expansion of knowledge of non-coding RNA biology has revealed critical roles in tumorigenesis processes (6). An increasing number of circRNAs have been found to participate in many pathological processes such as tumorigenesis through regulating genes expression at transcriptional, posttranscriptional, and translational (7–9). It is also reported that a variety of circRNAs are abnormally expressed in BC tissues or cell lines (10–13).

Since circRNAs have tissue and cell specificity, high abundance and stability, evolutionary species conservation and spread in various body fluids, exploring BC-related circRNAs as biomarkers or targets might be create new possibilities for the early diagnosis, effective treatment and prognosis evaluation of BC. In this review, we summarize the biological characteristics, functions, mechanisms of BC-related circRNAs, and finally discuss the potential applications as biomarkers, therapeutic targets.

## Biogenesis of circRNAs

CircRNAs are generated from precursor mRNA, and different from the canonical splicing of mRNA, circRNAs are produced by back-splicing process, which connects a 5′ splicing donor site with an upstream 3′ splicing receptor site to form a single chain covalent closed loop (9). The currently discovered circRNAs can be divided into three types: exonic circRNAs which contain exons only, intronic circRNAs which synthesized by introns, and exon-intron circRNAs which contain both exons and introns (9, 14).

However, the mechanisms for circRNAs biogenesis are not fully understood. Jeck et al. proposed two hypothetical models of exonic circRNAs formation: lariat-driven circularization and intron-pairing-driven circularization (15). Zhang et al. demonstrated that exon circularization is dependent on the complementary sequence of flanking introns (16). It was reported that microintrons containing splice sites and short reverse repeats can also form circRNAs (17). Subsequently, a new hypothetical model has been reported: RNA binding protein-mediated cyclization. ADAR1, muscle blind protein, and RNA-binding protein QKI are found to be critical in the formation of circRNAs (18–20).

## CircRNA Detection and Database for circRNAs Studies

In the current research, various techniques have been used detect and quantify circRNAs, including high-throughput RNA-seq, circRNA microarray, RT-PCR/qPCR, Northern blot, and some other technologies (21). High-throughout RNA-seq using next-generation sequencing, combine with depletion of ribosomal RNA to reveal the existence and quantity of circRNA (22). CircRNA microarray analysis uses circular ligation sequence specific probes combined with external nuclease linear RNA depletion to capture and quantify circRNAs at high sensitivity and specificity (15). Northern blot using the backsplice junction sequence specific probes and qRT‐PCR using divergent primers are used to verify and quantify limited known circRNAs (21).

Nowadays, with the continuous progress of circRNAs field, many circRNAs-associated databases have been built. The establishment of these databases makes it easier for researchers to analyze circRNAs’ information, regulatory networks, and role in diseases. The Circbase, CIRC pedia v2, and Deepbase 2.0 databases contain a large number of circRNAs and related detailed information about different species (23–25). The Circnet, Starbase v2.0, and circInteractome databases predict the circRNA-miRNA-gene network and interaction between circRNA and RNA-binding proteins (26–28). The CircRNADb and CSCD databases offer researchers the analysis of protein-encoding ability (29, 30). The CircRNADb database also contains genomic information, genome sequence, exon splicing, and references about circRNAs (29). The TRCirc database provides the regulatory information of circRNAs transcription (31). The CirclncRNAnet database provides a simple method for researchers to analyze sequencing results (32). The ExoRBase database and circRNA disease provides circRNAs existed in mutiple diseases or the related exosomes (33, 34). In addition to the above, there are some other related databases in constant establishment. However, due to the lack of standardized nomenclature, it is still difficult to search all the different databases for the same circRNA and compare these studies. In addition, database management and updates are still limited.

## Properties of circRNAs and The Application in Diseases

As a novel type of non-coding RNAs, circRNAs have circular structure, which is produced by unique back-splicing process with different mechanisms. Circular structure and different formation mechanism confer many unique characteristics to circRNAs such as high stability, evolutionary conservation, and tissues/cells specificity. These characteristics make it is possible to apply in the diagnosis, treatment and prognosis of diseases.

CircRNAs have been demonstrated to serve as miRNA sponges, regulate alternative splicing or transcription, bind to RNA binding proteins, perform rolling translation, encode proteins, as well as derive pseudo genes (4, 5, 35). CircRNAs are involved in many pathological processes, such as diabetes and its complications, nervous system disease, cardiovascular diseases as well as various tumors (5, 36–44).

In this review, we focus on the role, function and clinical application of circRNAs in the initiation, development, diagnosis, and prognosis of BC.

## CircRNAs in Bladder Cancer and Molecular Mechanisms

**Figure 1:**
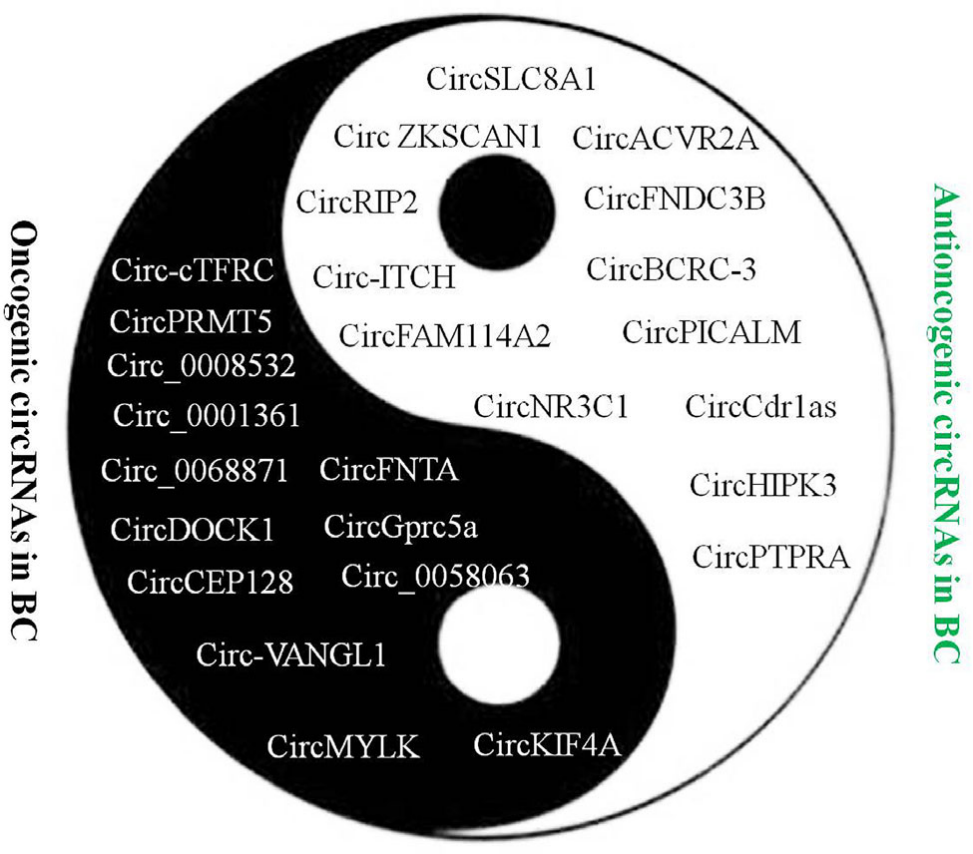
Oncogenic and antioncogenic circRNAs have been discovered in bladder cancer.

Increasing evidences indicated that circRNAs exert critical function in regulating BC occurrence and progression (**Figure 1**). More and more circRNAs have been discovered to regulate the proliferation, apoptosis, migration and invasion of BC cells. They played an important role in the occurrence and development of BC through various modes such as miRNA sponges and interaction with proteins (**Table 1**).

**Table 1:** Some examples of circRNAs that play an important role in the occurrence and development of BC.

CircRNAs	Sample	Dysregulation in GC	Potential function	Mechanism	References
CircPRMT5	BC tissues and paired tissues; BC cell lines; Animal models	Up-regulated	Correlated with tumor grade, EMT and poor survival rate	As a ceRNA for miR-107 to regulate SNAIL1/E-cadherin axis	Chen et al. (12)
Circ-cTFRC	BC tissues and paired tissues; BC cell lines; Animal models	Up-regulated	Correlated with tumor grade and poor survival rate	As a ceRNA for miR-107 to regulate TFRC expression	Su et al. (45)
Circ_0008532	BC tissues and paired tissues; BC cell lines; Animal models	Up-regulated	Correlated with tumorigenesis and invasion	As a sponge for miR-155-5p/miR-330-5p to regulate MTGR1/Notch pathway axis	Chen et al. (46)
Circ_0001361	BC tissues and paired tissues; BC cell lines; Animal models	Up-regulated	Correlated with tumor grade and invasion	As a sponge for miR-491-5p to regulate MMP9 expression	Liu et al. (47); Liu et al. (48)
Circ_0068871	BC tissues and paired tissues; BC cell lines; Animal models	Up-regulated	Promote the proliferation and migration of BC cells	Circ_0068871 up-regulated FGFR3 and activated STAT3 by targeting miR-181a-5p	Mao et al. (49)
CircFNTA	BC tissues and paired tissues; BC cell lines; Animal models	Up-regulated	Altered BC cells invasion and chemo-sensitivity to cisplatin	AR-mediated As a sponge for miR-370-3p or miRNA-451a then regulate KRAS or S1PR3	Chen et al. (50); Tian et al. (51)
CircDOCK1	BC tissues and paired tissues; BC cell lines; Animal models	Up-regulated	Promote the proliferation and migration of BC cells	As a sponge for miR-132-3p to regulate Sox5 expression	Liu et al. (52)
CircCEP128	BC tissues and paired tissues; BC cell lines; Animal models	Up-regulated	Promote the vitality, migration, and proliferation of BC cells	Regulating miR-145-5p via Myd88/MAPK or SOX11 signaling pathway	Sun et al. (53); Wu et al. (54)
CircGprc5a	BC tissues and paired tissues; Bladder CSCs; Animal models	Up-regulated	Affect the self-renewal and metastasis of bladder CSCs; related to clinical severity and metastasis of bladder cancer	CircGprc5a produced peptide and exerted its role in a peptide-dependent manner	Gu et al. (55)
Circ_0058063	BC tissues and paired tissues; BC cell lines; Animal models	Up-regulated	Promote the proliferation and migration of BC cells; inhibit of apoptosis	Acted as a sponge of miR-145-5p or miR-486-3p to regulate CDK6 or FOXP4 expression	Sun et al. (56); Liang et al. (57)
Circ-VANGL1	BC tissues and paired tissues; BC cell lines; Animal models	Up-regulated	Promote the proliferation, cell cycle, migration, and invasion of BC cells	Acted as a sponge of miR-605-3p which targeted VANGL1	Zeng et al. (58)
Circ-MYLK	BC tissues and paired tissues; BC cell lines; Animal models	Up-regulated	Accelerated the growth, angiogenesis, and metastasis of BC cells	Function as competing endogenous RNA for miR-29a to activate VEGFA/VER2 and downstream Ras/ERK pathway	Zhong et al. (59)
CircKIF4A	BC tissues and paired tissues; BC cell lines; Animal models	Up-regulated	Promote the proliferation and migration of BC cells	As a sponge for miR-375/1231 to regulate NOTCH2 expression	Shi et al. (60, 61)
CircRIP2	BC tissues and paired tissues; BC cell lines; Animal models	Down- regulated	Negatively associate with the grade, metastasis, and outcome of BC	Activating miR-1305/Tgf-β2/smad3 pathway	Su et al. (13)
CircZKSCAN1	BC tissues and paired tissues; BC cell lines; Animal models	Down- regulated	Negatively associate with survival, grade, metastasis, and recurrence	As a sponge for 1178-3p to regulate p21 expression	Bi et al. (10)
CircSLC8A1	BC tissues and paired tissues; BC cell lines; Animal models	Down- regulated	Negatively associate with the pathological stage, histological grade, invasion, and proliferation	Acted as a sponge of miR-130b/miR-494 to regulate PTEN expression	Lu et al. (11)
CircACVR2A	BC tissues and paired tissues; BC cell lines; Animal models	Down- regulated	Inhibit BC cell proliferation and metastasis	As a sponge for miR-626 to regulate EYA4 expression	Dong et al. (62)
Circ5912	BC tissues and paired tissues; BC cell lines; Animal models	Down- regulated	Negatively associate with grade, stage, metastasis, and overall survival time	Regulated TGF-β2 pathway	Su et al. (63)
CircFNDC3B	BC tissues and paired tissues; BC cell lines; Animal models	Down- regulated	Correlated with pathological stage, grade, proliferation, invasion, and overall survival rate	Acted as a miR-1178-3p sponge to suppress G3BP2/SRC/FAK pathway	Liu et al. (64)
CircITCH	BC tissues and paired tissues; BC cell lines; Animal models	Down- regulated	Inhibited the proliferation, invasion, and metastasis of BC cells	Acte*d* as a tumor suppressor by circ-ITCH/miR-17, miR-224/p21, PTEN axis	Yang et al. (65)
CircBCRC-3	BC tissues and paired tissues; BC cell lines; Animal models	Down- regulated	Inhibited the proliferation of BC cells	As a sponge for miR-182-5p to regulate p27 expression	Xie et al. (66)
CircFAM114A2	BC tissues and paired tissues; BC cell lines; Animal models	Down- regulated	Inhibited the migration, invasion, and proliferation of BC cells	Acted as a sponge of miR-762 to regulate ∆NP63 expression	Liu et al. (67)
CircPICALM	BC tissues and paired tissues; BC cell lines; Animal models	Down- regulated	Correlated with pathological stage, grade, metastasis, and overall survival rate	Acted as a sponge of miR-1265 to influence FAK phosphorylation	Yan et al. (68)
CircCdr1as	BC tissues and paired tissues; BC cell lines; Animal models	Down- regulated	Overexpression of Cdr1as inhibited proliferation, invasion and migration; induced the apoptosis and enhanced the cisplatin chemosensitivity of BC cells	As a sponge for miR-1270 or miR-135a to regulate APAF or p21 expression	Yuan et al. (69); Li et al. (70)
CircHIPK3	BC tissues and paired tissues; BC cell lines; Animal models	Down- regulated	Inhibited cell cycle progression and proliferation of BC cells	As a sponge for miR-27a-3p to regulate cyclin D1 expression	Li et al. (71); Xie et al. (72)
CircPTPRA	BC tissues and paired tissues; BC cell lines; Animal models	Down- regulated	Associated with prognosis, stage, and proliferation	As a sponge for miR-1270 to regulate KLF9 expression	He et al. (73)

### Oncogenic circRNAs in Bladder Cancer

Circ-cTFRC might be correlated with grade and poor survival rate of BC patients through circ-cTFRC/miR-107/TFRC axis. As known a sponge for miR-107, circ-cTFRC are up-regulated in BC tissues and cell lines, which related to grade, invasion, proliferation and poor survival rate. The expression of circ-cTFRC correlated with TFRC and negatively correlated with miR-107 in BC tissues and cell lines (45).

CircPRMT5 promoted metastasis of BC through sponging miR-30c to induce EMT. CircPRMT5 was up-regulated in BC tissues and associated with advanced clinical stage and worse survival of BC patients. Moreover, circPRMT5 is also up-regulated in serum and urine exosomes of BC patients, indicating that it may be significantly related to metastasis (12).

Circ_0008532 was revealed as miR-155-5p and miR-330-5p sponge and could regulate the capacity for invasive in BC cells through MTGR1/Notch pathway. Circ_0008532 is up-regulated in BC cells and tissues, which acted as an oncogene in BC, which may provide potential biomarkers and a therapeutic target for the treatment of BC (50).

A novel circRNA, circ_0001361 was recently found to act as oncogenic circRNA (47, 48). Circ_0001361 was highly expressed in BC tissues and cell lines, and it was positively correlated with grade, invasion, and poor overall survival. Mechanistically, circ_0001361 could directly interact with miR-491-5p to up-regulate MMP9, and MMP9 was verified to mediate circ_0001361-induced migration and invasion of BC cells (47).

Circ_0068871 was a circRNA derived from several exons of FGFR3, plays the role of oncogenes in the occurrence and development of BC, and serves as a potential biomarker (49). Circ_0068871 up-regulated expression of FGFR3 and activated STAT3 through sponging miR-181a-5p to promote cells proliferation and migration.

There are many other circRNAs (such as CircFNTA, circDOCK1, CircGprc5a, CircCEP128, Circ_0058063, Circ-VANGL1, CircRMYLK, and CircKIF4A) that play important roles in promoting BC, which could be used as potential clinical BC diagnosis and prognosis biomarkers (46, 51–60).

### Antioncogenic circRNAs in Bladder Cancer

Su et al. found that circRIP2 may serve as tumor suppressor in BC through sponge for miR-1305 (13). Higher circRIP2 expression negatively associates with the grade, metastasis, and outcome of BC patients. They found that circRIP2 sponge miR-1305 to accelerate BC progression by activating TGF-β2/smad3 pathway.

Circ ZKSCAN1 was markedly down-regulated in BC tissues and cells and with survival, tumor grade, pathological stage, and tumor recurrence (10). Overexpressed circ-ZKSCAN1 inhibited the proliferation, invasion, and metastasis of BC cells. They also demonstrated that circZKSCAN1 suppressed the aggressive biological behaviors of BC cells through up-regulates the expression of p21 by sponging miR-1178-3p.

CircSLC8A1 is a circRNA transcribed from gene SLC8A1. CircSLC8A1 was identified from RNA-sequencing data, might serve as potential tumor suppressor for BC (11). They found that circSLC8A1 was down-regulated in BC tissues and cells, which was negatively correlated with the stage and grade of BC. CircSLC8A1 acted as miRNA sponge to regulate the expression of the miR-130b/miR-494 target gene PTEN, which suppressed the migration, invasion, and proliferation of BC cells.

CircACVR2A might serve as potential suppressive factor for BC, which selected from RNA-sequence, was found to be decreased in BC tissues and cell lines (62). CircACVR2A acted as a miRNA sponge of miR-626 to regulate EYA4 expression and suppressed the proliferation, migration, and invasion of BC cells.

CircFNDC3B acted as a miR-1178-3p sponge to suppress G3BP2, which served as a novel tumor suppressive factor and potential target for new therapies in human BC. CircFNDC3B was dramatically down-regulated in BC tissues and correlated with grade, proliferation, migration, invasion, and overall survival rate (64).

Circ5912 played the role of tumor suppressor gene in BC by regulating TGF-β2. Circ5912 expression was significantly lower in BC tissues. Higher circ5912 levels associated with better clinical outcomes, including grade, stage, metastasis, and longer overall survival time (63).

There are many other anti-cancer circRNAs that play important roles in the diagnosis, treatment and prognosis of BC and may be used in clinical practice in the future (58, 65–74).

## CircRNAs as Diagnostic and Prognostic Biomarkers for Bladder Cancer

The high incidence, high mortality and poor prognosis of BC make new requirements for early diagnosis and the prognosis. CircRNAs have been shown to have great potential as cancer diagnostic and prognostic biomarkers. Firstly, circRNAs can be easily detected due to the high stability and abundance in various tissue of human. Secondly, many circRNAs expression are tissue specific and development stage specific, which has an important role in diagnosis and prognosis. In addition, circRNAs are also exists in serum, plasma, and other body fluids, which can be used for non-invasive detection (9, 75, 76).

The clinical value of circRNAs as diagnostic and prognostic biomarkers of BC has been explored in many studies (62, 77–79). Subsequently, through the correlation analysis of clinic pathological factors and prognosis and survival analysis, a set of potential circRNAs biomarkers appeared for the early diagnosis of BC and the prediction of recurrence and metastasis (**Table 2**).

**Table 2:** Analysis of clinical application of CircRNAs.

CircRNAs	Dysregulation in GC	Analysis of clinical application	References
Circ_0077837	Down-regulated	A promising biomarker for the early diagnosis, prognosis, and therapy of BC	Shen et al. (80)
Circ_0004826	Down-regulated	A promising biomarker for the early diagnosis, prognosis, and therapy of BC	Shen et al. (80)
Circ_0137439	Up-regulated	A promising biomarker for early diagnosis and prognostic assessment of BC	Song et al. (81)
Circ-cTFRC	Up-regulated	A potential marker of BC diagnosis or progression	Su et al. (45)
Circ5912	Down-regulated	A promising progression marker and a potential therapeutic target of BC	Su et al. (63)
Circ ZKSCAN1	Down-regulated	A potential progression marker and a prognostic factor of recurrence	Bi et al. (10)
CircSLC8A1	Down-regulated	A potential progression marker and a potential therapeutic target of BC	Lu et al. (11)
CircACVR2A	Down-regulated	A potential prognostic biomarker and therapeutic target of BC	Dong et al. (62)
CircFNDC3B	Down-regulated	A potential prognostic biomarker and therapeutic target of BC	Liu et al. (33)
CircPRMT5	Up-regulated	An exploitable therapeutic target of BC	Chen et al. (12)
Circ_0008532	Up-regulated	A potential therapeutic target of BC	Chen et al. (46)
circ_0001361	Up-regulated	A potential novel target for BC therapy	Liu et al. (47); Liu et al. (48)
Circ_0068871	Up-regulated	A potential marker of BC progression	Mao et al. (49)
Circ_0000285	Down-regulated	An independent prognostic assessment factor of BC	Chi et al. (82)

According to the detection of tissue and serum samples from BC patients and the controls, followed with clinic pathologic factors correlation analysis as well as prognostic and survival analysis, a set of potential circRNAs biomarkers are verified for BC diagnosis and prognosis. Circ_0077837 and circ_0004826 was significantly correlated with worse clinicopathological features and poor prognosis of BC patients. The area under the ROC curve of them was 0.775 and 0.790, respectively (80). Circ_0137439 was correlated with tumor stage, grade, lymph node status, and history of muscle-invasive BC. Urinary cell-free circ_0137439 could not only distinguish BC from normal controls, but also distinguish muscle-invasive BC from non-muscle-invasive BC. In addition, circ_0137439 in urine supernatant could be used as an independent prognostic indicator of recurrence-free survival and overall survival of BC patients (81). Circ_0000285 was significantly reduced in BC tissues and serum. Moreover, circ_0000285 was associated with tumor size (p < 0.001), differentiation (p < 0.001), distant metastasis (p = 0.004), TNM stage (p = 0.013), and lymph node metastasis (p = 0.038) (82). Higher circ5912 levels associates with better clinical outcomes, including grade (p = 0.008), metastasis (p = 0.016), and longer overall survival time (p = 0.0157) (63).

In conclusion, circRNAs play an important role in the occurrence and development of BC, which may be served as potential biomarkers for the diagnosis, prognosis, and therapy of BC.

## CircRNAs as Potential Therapeutic Targets for Bladder Cancer

As the roles in BC are gradually being clarified, circRNAs might be developed as potential therapeutic targets. Studies have proposed several strategies based on the function of circRNAs to treat BC. Exogenous up-regulation or down-regulation of related circRNAs to regulate miRNA may be the useful methods, and several of them have been applied. SiRNA or shRNA targeting a specific reverse splicing sequence of circRNAs was used to inhibit its expression (9, 83, 84). Stable circRNAs knockdown was generated using specific lentiviral short hairpin RNA (85–87). Some researchers have also tried to use the CRISPR/Cas9 system to achieve circRNAs knockout (88, 89). It was also reported that plasmids and lentiviral vectors used to increase the expression of circRNAs (88, 90). Overexpression vectors with short intronic repeat sequences also could increase the levels of circRNAs (17). In addition to exogenous regulation of circRNAs expression, endogenous regulation also has broad application prospects. However, there are still no relevant reports on how to control the process of endogenous cyclization.

Artificial circRNAs sponges targeting miRNAs may be a simple, effective, and convenient strategy for BC treatment. A large number of studies have found that circRNAs as miRNA sponges can inhibit the progression of BC (10, 11, 13, 62). These results suggested that BC can be treated by synthesizing related circRNAs sponges. Recently, synthetic circRNA presented a new treatment approach for critical global pathologies, including cardiovascular disease, hepatitis, esophageal carcinoma, and Gastric Carcinoma (91–94). These findings establish that the design and construction of highly efficient artificial circRNAs sponges represent a novel strategy in the treatment of cancer and other diseases.

Artificially controlled endogenous circularization may be another approach to apply circRNAs to the treatment of BC. On one hand, the “mRNA trap” can be used to isolate the translation initiation site of these abnormal mRNA to reduce the production of BC-related functional proteins (9, 95). On the other hand, using circRNAs to regulate the release and activity of BC-related proteins, or targeting coding circRNAs involved in tumorigenesis or development may be potential therapeutic methods (9).

## Challenges and Perspectives

CircRNAs are becoming an emerging field of tumor diagnosis and treatment research, but the experimental and clinical researches in BC still many unresolved. Firstly, the mechanism of cyclization and degradation of BC-related circRNAs is still unclear. Secondly, the number of BC-related circRNAs with clear biological functions and mechanisms of action is limited. Besides, under what conditions circRNAs play a cancer-promoting effect, and what conditions play a cancer-suppressing effect is not clear. In addition, it is not clear whether circRNAs can affect BC microenvironment cells and play a role in BC development.

Exosomes are nano-scale vesicles released by many cells, which can transfer signal molecules including circRNAs to recipient cells and regulate the progression of many diseases, including cancer. Exosomes might extremely extend circRNAs’ studies and applications. It is reported that exosomal circRNAs can be used as emerging tools for cancer diagnosis, prognosis and risk assessment (9, 61, 96, 97). Based on these studies, we believe exosomal circRNAs may be expected to become a promising biomarker and therapeutic tool for BC.

Noncoding RNA methylation modification in cancer is getting more and more attention from researchers. Recent studies have indicated that several circRNAs are closely related to the tumorigenesis of cancers *via* post-transcriptional methylation modificationsp (98–100). It provides a new research direction for us to explore the role of circRNAs in the occurrence and development of BC.

Nevertheless, the clinical application of circRNAs as drugs or targets in BC needs more detailed and complete experimental data such as safety, efficacy, and potential side effects. The systematic assessment including the cost, accuracy, repeatability, specificity, and sensitivity of circRNAs biomarkers in large samples is not sufficient. Furthermore, standardized methodology for circRNAs detecting in the process of clinical application is lacked. In addition, how to safely and effectively deliver circRNAs in vivo is also a problem to be solved. These challenges and deficiencies provide the direction for the follow-up research and technology development. We believe that with the deepening of research, the continuous progress of technology and the development of various aspects, these problems will eventually be solved.

## Conclusion

Through traditional/emerging biological methods and informatics technologies along with these further studies, more and more BC-related circRNAs and their physiological and pathological functions have been identified. In addition, many circRNAs have been found to have great potential to be diagnosis and prognosis biomarkers for BC as well as novel therapeutic targets or approaches. The knowledge of the precise mechanisms of circRNAs cyclization, degradation, localization, and function in BC is still limited in current stage. But, we believe that these limitations will eventually be resolved, these novel diagnosis and treatment strategies based on circRNAs will effectively serve BC clinical early diagnosis and treatment in the future.

## Author Contributions

ZL, HQ, and WX contributed to the conception and design of this review. ZL wrote the first draft of the manuscript. WG, SF, YZ, and LL wrote sections of the manuscript. All authors contributed to the article and approved the submitted version.

## Funding

This work was supported by grants from the National Natural Science Foundation of China (no. 81602883), Zhenjiang Key Laboratory of High Technology Research on Exosomes Foundation and Transformation Application (Grant SS2018003), Zhenjiang Social Development Guidance Project (No. FZ2019038), the Foundation for Excellent Young Teachers of Jiangsu University, and Research and Practice innovation program for graduate students of Jiangsu Province (No. KYCX20_3089).

## Conflict of Interest

The authors declare that the research was conducted in the absence of any commercial or financial relationships that could be construed as a potential conflict of interest.
